# Assessment of Worldwide Genetic Diversity of Siberian Wild Rye (*Elymus sibiricus* L.) Germplasm Based on Gliadin Analysis

**DOI:** 10.3390/molecules17044424

**Published:** 2012-04-12

**Authors:** Xiao Ma, Shiyong Chen, Xinquan Zhang, Shiqie Bai, Changbing Zhang

**Affiliations:** 1 Department of Grassland Science, Sichuan Agricultural University, Yaan 625014, China; Email: maroar@126.com (X.M.); chengshi8827@163.com (S.C.); zhangxq8@hotmail.com (X.Z.); 2 Sichuan Academy of Grassland Science, Chengdu 611731, China; Email: c.b.zhang@126.com

**Keywords:** *Elymus sibiricus*, genetic diversity, cluster analysis, gladin markers

## Abstract

*E. sibiricus* L., the type species of the genus *Elymus*, is a perennial, self-pollinating and allotetraploid grass indigenous to Northern Asia, which in some countries can be cultivated as an important forage grass. In the present study, eighty-six *Elymus sibiricus* accessions, mostly from different parts of Asia, were assayed by gliadin markers based on Acid Polyacrylamide Gel Electrophoresis to differentiate and explore their genetic relationships. The genetic similarity matrix was calculated by 47 polymorphic bands, which ranged from 0.108 to 0.952 with an average of 0.373. The total Shannon diversity index (H_o_) and the Simpson index (H_e_) was 0.460 and 0.302, respectively. Cluster analysis showed a clear demarcation between accessions from Qinghai-Tibetan Plateau, China and the others as separate groups. The clustering pattern was probably dependent on geographic origin and ecological adaptability of the accessions. The population structure analysis based on Shannon indices showed that the proportion of variance within and among the five geographic regions of the Northern Hemisphere was 55.9 and 44.1%, respectively, or 63.4 and 36.6% within and among six Chinese provinces. This distinct geographical divergence was perhaps depended on ecogeographical conditions such as climate difference and mountain distribution. The results of gladin analysis in this study are useful for the collection and preservation of *E. sibiricus* germplasm resources.

## 1. Introduction

*Elymus* L. is the largest genus in the tribe *Triticeae* and by various estimates has 150 species distributed in most temperate regions of the World [1]. As the type species of the genus *Elymus*, *E. sibiricus* L. (Siberian wildrye) is a perennial, caespitose, self-pollinating and allotetraploid grass (2n = 4x = 28) indigenous to Northern Asia, possessing the St and H genome [2]. Its natural geographic distribution extends from Sweden to Japan and even to parts of Alaska and Canada [3]. It usually grows on wet meadows, riverside sands, among open forest, on sunny or semi-shade slopes of mountains or valleys at altitudes from 1,000 up to 4,000 m. In dry or semidesert regions of Central Asia and Xingjiang province of Northwestern China, *E. sibiricus* usually serves as an important forage grass, and has been widely employed in establishing artificial grasslands to develop stock raising [4,5], due to its strong adaptability, excellent tolerance to drought and cold, high crude protein content, and good palatability. It has also played an important role in natural grassland restoration in Qinghai-Tibet Plateau of China as a pioneer grass species [5].

Knowledge of the genetic diversity in wild germplasm collections is central to the development of its effective conservation in genebanks, the insight of its evolutionary process and the efficient utilization in breeding strategies. Interest in the genetic structure of natural populations of grass species has been increased in the last few years owing to the necessity of broadening the knowledge of genetic variations in cultivated species. 

Gliadins, one of the main grain components of seed storage proteins in the tribe of *Triticeae*, are monomeric proteins of 30–78 kD with poor solubility in dilute salt solutions and good solubility in aqueous ethanol [6,7]. The studies carried out on seed storage proteins (prolamins) have suggested that their analysis could provide a measure of genetic diversity within and between populations of wheat, barley and other closely related species [8,9,10,11,12]. The great variability of these proteins, consequence of their neutral nature at evolution level, can substantially contribute to the analysis of evolutionary forces causing population genetic structure and diﬀerentiation [8]. Likewise, the storage proteins, especially gliadins on account of greater variability, could be used as ideal genetic markers since they are direct products of genetic differentiation [8,13]. All prolamin proteins in *Triticeae* are encoded by highly conserved multigenic families based on chromosomal locations, and the difference of copy number leads to gliadin polymorphism [14]. Phylogenetic analysis showed that α/β-gliadin genes from *E. sibiricus* were more homologous to those from other *Triticum* species [15]. This made it tremendously feasible to utilize gliadin to perform genetic researches in *Elymus* species like genus *Triticum* and *Hordeum*. As a matter of fact, gliadin analysis based on Acid Polyacrylamide Gel Electrophoresis (A-PAGE) has been successfully applied in *Elymus* genus, mainly *E.sibiricus* and *E. nutans*, in which considerable interspecific or intraspecific variation were showed [16,17,18]. Besides, some DNA molecular markers including simple sequence repeats (SSR) [19], inter-simple sequence repeats (ISSR) [20], and sequence-related ampliﬁed polymorphism (SRAP) [21] has been reported in *E. sibiricus*. Despite fewer detectable loci as compared to DNA molecular markers, gliadin markers still have great application potential in identification genotypes and characterization of genetic relationships of plant germplasm, because of its simplicity, rapidity and high repeatability.

For an insight of the electrophoretic patterns variation of gliadins of *E. sibiricus*, however, wider collections need to be studied, analyzing the degree of diversity of the gliadins between different wild accessions and cultivated materials, and examining the relationship of this diversity with the geographical, ecological and/or climatic conditions of the places where samples are collected. A group of worldwide accessions of *E. sibiricus*, selected by their differences in terms of ecogeographic origin, formed the material examined in the present work. The aim of this study is to understand genetic variation and structure of eighty-six *E. sibiricus* accesssion that were collected worldwide from the main distribution areas including Western China, Mongolia, Central Asia, Siberia, *etc*., using a standard wheat variety ‘Marquis’ for A-PAGE analysis as control. Through an understanding of genetic diversity in *E. sibicus* from different regions, it will be helpful to produce a more efficient collection and preservation strategy for different regions and populations, and to maximize the use of germplasm resources.

## 2. Results and Discussion

The following 86 accessions of *E. sibiricus* were used in this study: 10 from Mongolia, four from Kazakhstan, three from North America, 16 from Russia, and 53 from China (Table 1). The great majority of these accessions were wild materials. Additionally, all of Chinese accessions except those from Inner Mongolia were collected from the Qinghai-Tibet Plateau including Qinghai, Tibet, Sichuan and Gansu. 

**Table 1:** Description of the 86 *E. sibiricus* accessions used in this study.

Source	Accession codes
Mongolia	PI610850, PI610857, PI610860, PI610862, PI610866, PI610876, PI610886, PI628726, PI634230, PI634231
Kazakhstan	PI314619, PI598773, PI598778, PI598788
Alaska, US	PI372696, PI348916
British Columbia, Canada	PI372541
Siberia, Russia	PI326266 **, PI628699, PI598478, PI369236, PI611013, PI598800, PI634228, PI406467 **, PI611020, PI315427, PI598789, PI345600 **, PI345599 **, PI326267 **, PI315428, PI598774
Xinjiang, China	Y0811, Y004, Y0860, Y0771, Y0766, Y0760, Y2027, Y0822, Y0473, Y013, Y0877, Y1914, Y2024, Y1823, Y0909, Y0486, Y005, PI628677, PI499462
Inner Mongolia, China	PI499453, PI499455, PI499457 *, PI499459 *
Sichuan, Qinghai-Tibet Plateau, China	205124, 204145, 204155, 205189, 205201, 205179, 204081, 204089, 204119, 205215, 205156, 205226, Chuancao No.2 **, Chuancao No.1^**^, 205171, 205172
Gansu, Qinghai-Tibet Plateau, China	Y2906, PI499456 *, PI499458 *, PI499460 *, PI499461, PI531671, PI636676, 204451
Qinghai, Qinghai-Tibet Plateau, China	PI504462, PI504463, PI531669, 204441
Tibet, Qinghai-Tibet Plateau, China	204404, 204251

* = cultivated material, ** = cultivar, and the rest = wild material.

Since quite a few accessions lacked information about concrete location, altitude, or habitats, only country origin and codes were listed in Table 1. Nevertheless, these countries or regions have their own unique ecogeographic conditions, which could provide the good reference information for the present study.

### 2.1. Polymorphism of Gliadin Bands

**Figure 1:**
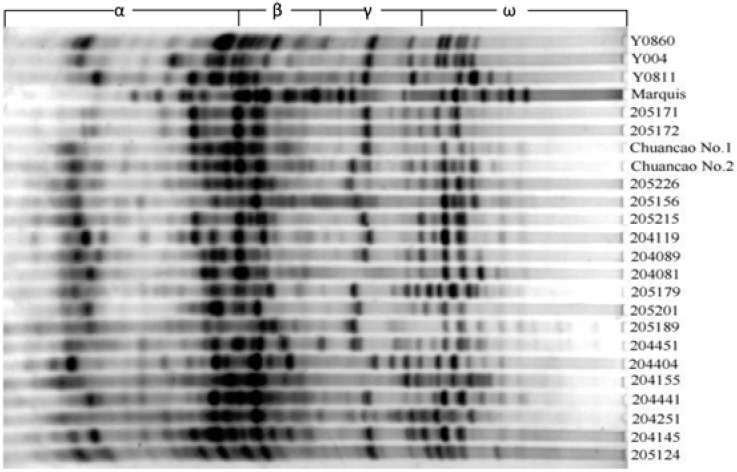
Gliadin patterns of parts of accessions of *Elymus sibiricus* after acid polyacrylamide gel electrophoresis (A-PAGE). The places of α, β, γ and ω-gliadins are indicated.

Using the A-PAGE procedure with the wheat variety ‘Marquis’ as a control, a total of 52 electrophoretic bands were detected in 86 accessions of *E. sibiricus*, which were fractioned into α-, β-, γ-, and ω-zone with 10, 9, 12, and 21 bands, respectively (Figure 1). The average number of scoreable bands per zone was 13, and the average number of polymorphic bands was 11.75. In the 52 bands, only five are common to all materials, the percentage of polymorphic bands (*PPB*) of which, were 90.4%. To measure the genetic content of accessions for four electrophoretic zones, we used the Shannon index (H_o_), which is essentially a sum of frequency of occurrence of events (the gliadin bands in this case) (Table 2). The Shannon diversity index (H_o_) was highest in γ-zone (0.557), followed by β-zone (0.472), α-zone (0.431), and ω-zone (0.401). The high values of the total Shannon diversity index (H_o_ = 0.460) confirmed that gliadin markers are a very suitable tool for large-scale screening of *E. sibiricus* germplasm. The Simpson index (H_e_) and the Shannon index were changed with a similar tendency. In addition, the high level of variation found in this study is consistent with the variability observed in morphological traits. 

**Table 2:** The genetic diversity indexes among 86 accessions of *E. sibiricus*.

Zones	Total bands	Polymorphic bands	Po1ymorphism percentage (%)	Shannon index (H_o_)	Simpson index (H_e_)
α	10	10	100	0.431	0.274
β	9	7	77.8	0.472	0.318
γ	12	12	100	0.557	0.376
ω	21	18	85.7	0.401	0.253
Total	52	47	90.4	0.460	0.302

The high polymorphism detected in the present investigation could be comparable to previous reports about *E. sibiricus*, such as gliadin (PPB = 92.86%) [16], ISSR (PPB = 77.20%) [20], SRAP (PPB = 86.48%) [21], and SSR (PPB = 86.44%) [19], of which gliadin analysis of *E. sibiricus* used 52 accessions from Qinghai-Tibet Plateau [16], and ISSR analysis focused on native populations from a narrow distribution area in northwestern plateau of Sichuan province of China [20]. The high level of genetic variability among the studied accession worldwide probably corresponds to the diversity of geographical origin or ecological conditions. 

### 2.2. Genetic Similarities and Cluster Analysis

The genetic similarities (GS) among 86 accessions were estimated by Jaccard coefficient based on gliadin markers. The GS for the 86 accessions ranged from 0.108 to 0.952 with a mean of 0.382. The lowest value of GS was 0.108, which was observed between accession PI598800 (Russia) and 205124 (Sichuan, China), while the highest GS (0.952) was found between accession PI372696 and PI348916, both collected from Alaska, USA. The average of GS among North America accessions was 0.859, while it was 0.636, 0.494, 0.418 and 0.391 for Mongolia, Kazakhstan, Russia and China. In the China group, the highest average of GS (0.555) was found among accessions from Qinghai, followed by Inner Mongolia (0.554), Xinjiang (0.514), Tibet (0.483), Gansu (0.454) and Sichuan (0.452). 

**Figure 2:**
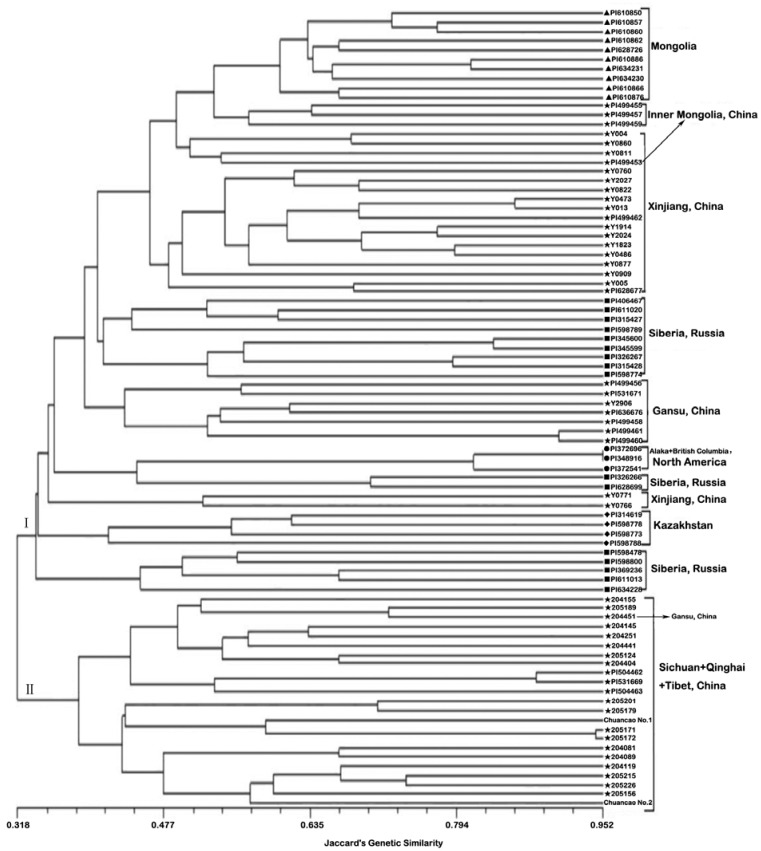
Dendrogram of 86 accessions derived from an UPGMA cluster analysis based on based on Jaccard’s similarity. Symbols show North America (●), Kazakhstan (◆), Mongolia (▲), Russia (■), and China (★).

The UPGMA dendrogram resolving the genetic relationship among 86 accessions based on gliadin markers is presented in Figure 2. The correlation between the cophenetic value matrix and the original similarity coefficient matrix was high (r = 0.826) indicating a good fit of the cluster analysis performed. The dendrogram allowed two main clusters to be distinguished. The first cluster (I) contained 63 accessions: three from North America, four from Kazakhstan, ten from Mongolia, sixteen from Russia, and thirty from China (Xinjiang, Gansu and Inner Mongolia). It is noteworthy that North American accessions showed extremely low genetic variation (mean GS = 0.859) and grouped with Eurasian accessions in cluster I. That is because Siberian wild rye is most likely a non-native grass that was recently introduced to Alaska and northwestern Canada from Russia or central Asia and they are often located in areas historically associated with human travel, agricultural experimentation, and revegetation after fire [22]. The second cluster (II) consisted of 23 accessions collected from Qinghai-Tibetan Plateau, China. Sixteen of which was distributed in Sichuan, four in Qinghai Province, two in Tibet Province, and one in Gansu Province. 

According to the dendrogram displaying the genetic relationships among all the accessions, the distribution of the accessions within the clusters has apparent relation with the ecological or geographic origin. Geographic origin of accessions might be expected to represent different macro-environments and thus explain much of genetic variation among accessions from different regions [23]. Nevo (1998) emphasized the role of ecological factors in determining the extent and distribution of genetic diversity [24]. Genetic diversity in *E.* *sibiricu*s as revealed by ISSR markers suggests that geographic isolation strongly inﬂuenced the evolution of the populations [20]. Thus, it is probable that this difference between cluster Ι and ΙΙ relies on ecogeographic adaptability of *E. sibiricus* accessions, since Qinghai-Tibet Plateau is geographically isolated from the rest of the world by several of the world’s largest mountain ranges. However, in Cluster Ι the sub-cluster groupings based on gliadin data were generally not strongly associated with particular geographic origins (e.g., accessions from Russia and Xinjiang, China), resulting from lack of exact definition of ecological zones for most accessions in this study, and the various selection forces that tended to produce the genetic heterogeneity under the different small niches, or gene mutation, natural factors and human activities. Moreover, genetic exchanges (*i.e.*, pollen crossbreeding) among different accessions in the progress of reproduction also compromise origin data.

### 2.3. Genetic Differentiation of Geographic Groups

The gliadin bands frequency detected using A-PAGE analysis were calculated and used in estimating Shannon diversity (H_o_) with geographic group types. The North American group exhibited the lowest variabilities within group, but consisted of the smallest group size (two accessions). Both the Chinese and Russian groups exhibited high variabilities within group (Table 3). For four Chinese sub-groups, Qinghai sub-group exhibited the lowest within group variabilities, whereas Sichuan and Xinjiang sub-groups exhibited high within group variabilities (Table 4). An assessment of the proportion of diversity present within groups, H_within_ = H_pop_/H_sp_, compared with that between groups, H_between_ = (H_sp_ − H_pop_)/H_sp_, indicated that intra-group diversity component accounted for 55.9% of the total diversity, while 44.1% was due to diversity between groups. This result showed that intra-group diversity was higher than inter-group diversity. Similar result was observed among four Chinese sub-groups as well. In addition, Shannon indices also revealed that 63.4% and 36.6% variation accounted for within and among six Chinese geographical subgroups, respectively (Table 4). These results was consistent with the previous researches of *E. sibiricus *germplasm collections, by Gliadin marker [16] and SRAP marker [21], which detected 68.17, and 65.29% variation within geographical groups, respectively. Typically, self-pollinating species maintain relatively more of their genetic variation among populations than out-crossing species [25]. Each accession could be regarded as a distinct genotype as a result of the mating pattern of autogamy. Consequently, a higher variation exists within geographic groups consisting of different accessions. Since there is a signiﬁcant geographical pattern of variarion distribution of *E. sibirucs*, it is clear that great importance was attached to the ecological or geographic diversification of germplasm collections as well as the sample size in order to maximize the use and conservation of genetic resources.

**Table 3:** Shannon diversity indices for five geographic groups.

Shannon Index	North America	Russia	Mongolia	Kazakhstan	China
H_o_	0.053	0.340	0.205	0.240	0.446
H_pop_ = Σ H_o_/n	0.257				
H_sp_	0.460				
H_within_ = H_pop_/H_sp_	0.559				
H_between_ = (H_sp_ − H_pop_)/H_sp_	0.441				

**Table 4:** Shannon-weaver diversity indices for six Chinese geographic sub-groups.

Shannon Index	Xinjiang	Inner Mongolia	Gansu	Sichuan	Qinghai	Tibet
H_o_	0.316	0.201	0.356	0.374	0.280	0.173
H_pop_ = Σ H_o_/n	0.283					
H_sp_	0.446					
H_within_ = H_pop_/H_sp_	0.634					
H_between_ = (H_sp_ − H_pop_)/H_sp_	0.366					

Accessions from China (H_o_ = 0.447) and Russia (H_o_ = 0.340) contain the highest level of gliadin variation, whereas the lowest H_o_ value was observed in the accessions from North America. Significant correlation was found between genetic diversity and sampling size in the previous study [26,27]. Hence, too small sample size of geographic groups could lead decrease of assessed H_o_, especially when most of the accessions in a group (e.g., North America group) were collected from a narrow area. 

## 3. Experimental

### 3.1. Plant Materials

A total of 86 accessions of *Elymus sibiricus* obtained from the National Genetic Resources Program (USDA), the Triticeae Research Institute (Sichuan Agricultural University, China), and Sichuan Academy of Grassland Science (Sichuan, China), were analysed in this study (Table 1). The passport data of these accessions indicate that they were collected in China, Russia, Kazakhstan, Mongolia and North America. Twenty bulked grains for each accession were analysed to detect farthest genetic variance within each accession. 

### 3.2. Acid Ployacrylamide Gel Electrophoresis (A-PAGE)

Embryo-less seeds crushed into a fine powder were used to extract the endosperm storage proteins. Gliadins were extracted with 70% (v/v) ethanol and separated by Acid-PAGE at 10% (C: 3.84%) according to ISTA’s standard method [28]. Canadian wheat cultivar Marquis was included on each gel as standard check for interpretation of relative mobilities of gliadin bands. The gel was divided in four zones (α-, β-, γ-, and ω-gliadins) by the distance to the cathode according to the position established for other Triticeae [29]. The different bands present in the gel were classified according to the zone of the gel and numbered consecutively. 

### 3.3. Data Analysis

In the gliadin gel profiles the presence of a gliadin band was coded as ‘1’, whereas the absence of a band was coded as ‘0’.Total genetic diversity was estimated using the Shannon index as *H_o_* = −Σπ lnπ, where π is the frequency of a band across all the samples [30]. *H_o_* can be calculated and compared for the four zones of gliadins (α, β, γ and ω) separately. Shannon index within a subset of data was calculated as *H_sp_* = −Σπ_i_ lnπ_i_, where π_i_ is the frequency of a band within a subset. The average diversity between different groups was calculated as *H_pop_* = 1/*n* Σ *H_o_*, where H_pop_ is the average group diversity over *n* groups. The proportion of diversity present within groups, *H_wintin_* = *H_pop_*/*H_sp_*, can thus be compared with variation between groups, *H_beteeen_* = (*H_sp_* − *H_pop_*)/*H_sp_*. The Simpson index (*i.e.*, the expected heterozygosity, H_e_) was calculated as He = 1 − Σ*p_i_*^2^, where *p_i_* is the band frequency of the *i*th allele [31]. The binary data were analyzed using qualitative routine to generate Jaccard’s genetic similarity coefficient (GS) [32]. Based on the GS matrix, a dendrogram was constructed using the UPGMA (unweighted pair group method of arithmetic averages) method by NTSYS-pc software [33,34]. The matrix correlation (cophenetic correlation) between the original GS matrix and the corresponding cophenetic matrix was calculated to test the goodness of fit of a tree matrix and its associated dendrogram to the original distance matrix by the Mantel test [35]. 

## 4. Conclusions

In summary, the genetic diversity analyses indicated that a high level of genetic variance existed among the studied *E. sibiricus* accessions. The dengrograms constructed by UPGMA method indicated that the genetic distance among accessions was related to the origin of these accesssions. And a clear demarcation was found between accessions from Qinghai-Tibetan Plateau of China and the other geographic areas as separate groups. This was confirmed by population structure analysis, which showed that the inter-group component accounted for 55.9% of the total diversity, while 44.1% was due to diversity within geographic groups. Thus, great importance should be attached to the ecogeographic diversification of germplasm collections as well as the enlargement of sampling size in order to maximize the use and conservation of genetic resources. All of the results of our study also showed that gliadin marker technique is suitable for genetic diversity analyses of *E. sibiricus* resources. 
